# National Planetary Health learning objectives for Germany: A steppingstone for medical education to promote transformative change

**DOI:** 10.3389/fpubh.2022.1093720

**Published:** 2023-02-16

**Authors:** Katharina Wabnitz, Eva-Maria Schwienhorst-Stich, Franziska Asbeck, Cara Sophie Fellmann, Sophie Gepp, Jana Leberl, Nikolaus Christian Simon Mezger, Michael Eichinger

**Affiliations:** ^1^Chair of Public Health and Health Services Research, Institute for Medical Information Processing, Biometry and Epidemiology, Ludwig-Maximilians-Universität München, Munich, Germany; ^2^Centre for Planetary Health Policy, Berlin, Germany; ^3^Department of General Practice and Family Medicine, University Hospital Würzburg, Würzburg, Germany; ^4^Teaching Clinic of the Faculty of Medicine and Institute of Medical Teaching and Medical Education Research, University Hospital Würzburg, Würzburg, Germany; ^5^German Alliance on Climate Change and Health, Berlin, Germany; ^6^Charité—Universitätsmedizin Berlin, Corporate Member of Freie Universität Berlin and Humboldt-Universität zu Berlin, Berlin, Germany; ^7^Health for Future Cologne, Cologne, Germany; ^8^Institute of Medical Epidemiology, Biostatistics and Informatics, Martin-Luther-University Halle-Wittenberg, Halle (Saale), Germany; ^9^Department of Global Public Health, Karolinska Institutet, Stockholm, Sweden; ^10^Center for Preventive Medicine and Digital Health, Medical Faculty Mannheim, Heidelberg University, Heidelberg, Germany; ^11^Division of Pediatric Epidemiology, Institute of Medical Biostatistics, Epidemiology and Informatics, University Medical Center of the Johannes Gutenberg-University, Mainz, Germany

**Keywords:** climate change, curriculum development, education for sustainable healthcare, medical education, Planetary Health, Planetary Health Education, transformative education

## Abstract

Physicians play an important role in adapting to and mitigating the adverse health effects of the unfolding climate and ecological crises. To fully harness this potential, future physicians need to acquire knowledge, values, skills, and leadership attributes to care for patients presenting with environmental change-related conditions and to initiate and propel transformative change in healthcare and other sectors of society including, but not limited to, the decarbonization of healthcare systems, the transition to renewable energies and the transformation of transport and food systems. Despite the potential of Planetary Health Education (PHE) to support medical students in becoming agents of change, best-practice examples of mainstreaming PHE in medical curricula remain scarce both in Germany and internationally. The process of revising and updating the Medical Licensing Regulations and the National Competency-based Catalog of Learning Objectives for Medical Education in Germany provided a window of opportunity to address this implementation challenge. In this article, we describe the development and content of national Planetary Health learning objectives for Germany. We anticipate that the learning objectives will stimulate the development and implementation of innovative Planetary Health teaching, learning and exam formats in medical schools and inform similar initiatives in other health professions. The availability of Planetary Health learning objectives in other countries will provide opportunities for cross-country and interdisciplinary exchange of experiences and validation of content, thus supporting the consolidation of Planetary Health learning objectives and the improvement of PHE for all health professionals globally.

## 1. Introduction

The anthropogenic climate crisis and the transgression of other planetary boundaries including biodiversity loss, aberrant biogeochemical flows and pollution are the greatest threats to public health in the twenty-first century (1). Adverse health effects include increased morbidity and mortality due to extreme weather events such as floods and heat waves, changing patterns of vector-borne diseases, altered prevalences of non-communicable conditions like asthma and other atopic diseases and adverse effects on mental health (2). The interdependence between the climate and ecological crises, societal, political, and economic systems as well as health and wellbeing are at the core of the emerging concept of Planetary Health, defined as “the health of human civilization and the state of the natural systems on which it depends” (3). Mirroring this interdependence, most adaptation and mitigation policies including the transformation of transport, energy, and agri-food systems are associated with substantial health co-benefits by, *inter alia*, reducing air pollution, increasing physical activity, and improving nutrition.

Physicians play an important role in adapting to and mitigating the climate and ecological crises and thus in attenuating adverse health effects. This includes, but is not limited to, addressing risk factors associated with the climate and ecological crises in medical histories and anticipatory guidance, building expertise in the treatment of climate-sensitive health conditions and implementing policies to decarbonize healthcare systems. The contribution of physicians to adaptation and mitigation is based on a professional ethos emphasizing their responsibility for the wellbeing of individuals and populations now and in the future (4), high levels of trust among the general public (5, 6) and their role as advocates for health-promoting climate policies (7). Planetary Health Education (PHE) aims at equipping physicians with knowledge, attitudes, values, and skills to fully harness this potential. To strengthen the role of physicians as change agents in healthcare institutions and other sectors of society, PHE moreover stimulates the development of leadership attributes, confidence and systems thinking skills (8–10). While the urgency to integrate PHE into medical curricula has been frequently expressed (10–12), best-practice examples of mainstreaming PHE in medical curricula remain scarce both in Germany and internationally (13–16). To date, PHE in medical schools is mostly limited to student-driven extra-curricular lecture series and elective courses (17), with a majority of courses only available in English and targeting healthcare professionals from the global north (18).

In Germany, the National Competency-based Catalog of Learning Objectives for Medical Education (hereinafter referred to as *National Catalog of Learning Objectives*; *Nationaler Kompetenzbasierter Lernzielkatalog Medizin*, NKLM), first published in 2015 by the Association of Medical Faculties in Germany, is currently refined in a nationwide multi-stakeholder process. It consists of a mandatory core curriculum and is accompanied by several non-mandatory but thematically cross-cutting chapters that can be covered by medical schools. Paralleling the refinement of the National Catalog of Learning Objectives, the German Medical Licensing Regulations are currently updated. After approval by the Federal Council, both documents will provide the legal foundation for the structure and content of medical education in Germany. It is currently anticipated that both documents will go into effect in 2025 and will stimulate changes to the curricula of all medical schools such as strengthening primary and outpatient care and enabling clinical electives in local public health departments. The update of the Medical Licensing Regulations and the National Catalog of Learning Objectives constituted a unique window of opportunity to address the lack of PHE in German medical curricula by developing national Planetary Health learning objectives. Given their impact on curriculum design, we anticipate that the learning objectives will stimulate the development and implementation of innovative teaching, learning and exam formats in medical schools. Further background information on medical education in Germany and current reform processes is presented in Appendix 1 in the Supplementary material.

In this article, we describe the development and content of national Planetary Health learning objectives that adhere to the structural requirements of the National Catalog of Learning Objectives. We hope that the catalog of learning objectives will inspire similar activities in other (health-related) disciplines and countries.

## 2. Iterative development of national Planetary Health learning objectives

The development of a catalog of national Planetary Health learning objectives proceeded iteratively. Initially, an *ad hoc* task force comprised of seven medical students and two early career health professionals with expertise in PHE were invited by the Association of Medical Faculties in Germany in 2020 to develop a set of competency-based Planetary Health learning objectives for the non-mandatory part of the National Catalog of Learning Objectives. Given that no widely used Planetary Health learning objectives were available in 2020, the task force developed an initial set of learning objectives as well as superordinate competencies and sub-competencies based on their expertise in PHE including the development and implementation of Planetary Health elective courses. Based on this initial set, we iteratively revised, restructured, and expanded the learning objectives in five steps.

First, to improve the structure of the initial set of learning objectives, the taskforce—now consisting of four medical students and three early career health professionals with expertise in PHE—structured the learning objectives and the superordinate competencies and sub-competencies into different levels. Guided by prior work in medical education research (8), we applied the following levels of learning: (1) knowledge, (2) values and attitudes, and (3) transformative skills (Table 1).

**Table 1:** Competencies and sub-competencies in the catalog of national Planetary Health learning objectives and their alignment with the levels of learning presented by Frenk et al. (8).

**Competencies and sub-competencies**	**Levels of learning by Frenk et al**.
**PH 1. Graduates demonstrate foundational knowledge about core areas of Planetary and Global Health**.	**Knowledge**
PH 1.1. They describe anthropogenic environmental changes and demonstrate understanding of associated health effects.	
PH 1.2. They describe core concepts and stakeholders in Planetary and Global Health.	
**PH 2. Graduates reflect on their responsibility to maintain and foster health and the natural and societal systems on which it depends and demonstrate relevant competencies**.	**Values and attitudes**
PH 2.1. They describe areas requiring transformative change to enable health within planetary boundaries and identify concepts and actors to implement transformative change processes.	
PH 2.2. They reflect on core ethical principles of Planetary and Global Health and their role as future physicians.	
PH 2.3. They demonstrate intercultural competencies based on reflections on their own cultural, social, economic and educational background and professional position.	
**PH 3. Graduates describe and demonstrate skills to stimulate and implement transformative change in healthcare and other sectors of society**.	**Leadership skills**
PH 3.1. They demonstrate the capability to implement transformative change processes and to establish necessary preconditions.	
PH 3.2. They demonstrate skills for implementing transformative change processes in inter- and transdisciplinary settings.	
PH 3.3. They describe approaches to implement transformative change processes in healthcare systems and patient care.	

Second, to improve the comprehensiveness of the initial set of learning objectives, we expanded the set based on seminal contributions to the emerging field of Planetary Health and PHE. Two members of the task force independently mapped the learning objectives against the *12 Cross-cutting principles of planetary health education*, a set of foundational principles proposed to guide teaching in the field of Planetary Health (Table 2) (19). In addition, one member of the task force reviewed three additional documents, (1) the first Planetary Health textbook (20), (2) the Report of the Rockefeller Foundation–Lancet Commission on Planetary Health (3) and (3) the 2019 Report of the Lancet Countdown on Health and Climate Change (21). Based on gaps identified during the mapping and review, we expanded the initial set of learning objectives, resulting in a refined catalog comprised of three overarching competencies, eight sub-competencies and 31 learning objectives. To increase practical relevance and to facilitate the application of the Planetary Health learning objectives in curriculum development and teaching, we identified several health-related examples for each learning objective (see last column in Appendix 2 in the Supplementary material).

**Table 2:** Mapping of Planetary Health learning objectives against the *Twelve cross-cutting principles for Planetary Health Education* (19).

**Twelve cross-cutting principles for Planetary Health Education**	**Learning objectives**
1. A planetary health lens Many global challenges come into sharper focus when they are viewed with the idea of planetary health in mind. Equipping students with what we have called a planetary health lens will enable them to have an understanding and appreciation of the crucial linkages, cause–effect relationships, and feedback loops between environmental change and human health. Through this lens, students will be able to recognize and explore how human stewardship of the Earth is a primary determinant of future population health.	PH 1.1.1 PH 1.1.2 PH 1.1.3 PH 1.1.4 PH 1.1.5 PH 2.1.1 PH 2.1.2
2. Urgency and scale The field of planetary health is driven by the scale of environmental change, its effects on human health, and the urgency with which the global population must respond. Students should be able to examine the complexity of interactions between the geographical scale, temporal scale, socioeconomic factors, and political and cultural context that shape specific challenges to and potential solutions for sustainable human health outcomes.	PH 1.1.2 PH 1.1.3 PH 1.1.4 PH 1.2.1 PH 1.2.2
3. Policy Planetary health is intrinsically policy oriented. By quantifying the human health effects of anthropogenic environmental changes and communicating them to stakeholders at many levels, collaborative work can be done across sectors to identify policies and practices, both local and global, to protect and improve the health of global populations. A familiarity with the evidence gaps and policy applications of planetary health research, and an appreciation for agencies at the individual and community level are key for a meaningful and context-specific translation of research into policy and action.	PH 1.2.6 PH 1.2.7 PH 2.1.1 PH 2.1.2 PH 2.1.4
4. Organizing and movement building Students should develop an understanding of the role that organizing in the community and movement building has in the political process both locally and globally. They should have an appreciation for the influence of a so-called bottom-up approach to policy change, and that the capacity to mobilize and manage resources and people power is key when considering solutions to challenges in planetary health.	PH 2.1.4 PH 2.2.2 PH 3.1.1 PH 3.1.3 PH 3.2.3 PH 3.3.1
5. Communication Challenges in planetary health are complex, spanning different disciplines, sectors, geographical regions, cultures, and scales; therefore, effective and meaningful communication across these arenas is needed, with a focus on translating planetary health science. Students should develop an understanding of the variety of communication methods available and how to select the best suite of tools as they work to convey the challenges and solutions of planetary health to diverse audiences. An appreciation for the importance of listening as a part of effective communication is vital.	PH 3.1.1 PH 3.1.3 PH 3.2.1 PH 3.2.2
6. Systems thinking and transdisciplinary collaborations An understanding of planetary health necessitates engaging with many disciplines and stakeholders to understand and propose solutions to complex challenges. Thus, the incorporation of systems thinking and knowledge integration into curricula is essential to better equip students to collaborate across disciplines and develop sustainable solutions for the challenges of planetary health that overcome existing gaps in research design and associated policy development.	PH 1.1.1 PH 1.2.3 PH 1.2.7 PH 2.1.1 PH 2.1.2 PH 2.1.3 PH 2.1.4 PH 3.1.1 PH 3.1.3
7. Inequality and inequity Understanding the differences between equality and equity in theory and practice, and concepts of marginalization, vulnerability, resilience, and who benefits and is harmed in a given scenario, is a core objective of planetary health teaching. Since the effects of environmental change on human health are heterogeneous and mediated by factors such as geographical scale, temporal scale, socioeconomic factors, and political and cultural context, students should think critically about whose health is at stake and how it is measured.	PH 1.1.5 PH 1.2.1 PH 1.2.2 PH 2.3.3 PH 2.3.4
8. Bias Students should be able to think critically about whether political, social, or economic dynamics could be driving the presentation and perceptions of environmental change and the resultant health effects. They must learn to identify potential biases in planetary health research and be aware of the vested interests of different stakeholders both in support of and against the factors that affect the connection between environmental change and human health.	PH 1.2.3 PH 3.1.2
9. Governance Governance is the high-level strategy used by a leader or leadership team in their processes of decision making and implementation. It is the ability to turn capacity into action and generating the capacity when it does not exist. Governance requires dealing with institutional issues, managing political interests, and making leadership more effective. Students should understand and be able to provide some examples of how challenges in planetary health can be created or aggravated by the failures of governing bodies to cooperate across populations, regions, and boundaries, especially where effective cooperative mechanisms are not yet established.	PH 1.2.5 PH 1.2.6 PH 1.2.7 PH 3.2.3
10. Unintended consequences Students should appreciate that surprising and unexpected consequences of environmental change, both positive and negative, are inevitable. Students should understand the role and predictive limitations of impact assessments and recognize that how the Earth's changing biophysical conditions affect human health will continue to be a surprise. This systemic uncertainty requires a shift in government, corporate, and community mindsets to allow for increased adaptive capacity, and an emphasis on programmes that increase socioecological competence, community resilience, and sustainability.	PH 1.1.4 PH 1.1.5 PH 1.2.5 PH 2.1.2 PH 2.3.1 PH 2.3.2
11. Global citizenship and cultural identity A global citizen is someone who sees themselves as part of the international community and whose actions help define the community's values and practices. If students can realize their own cultural identities and recognize their inherent membership in both their local and global communities, they have the opportunity to help define the values and practices of the next generation to positively affect those communities.	PH 2.2.1 PH 2.2.2 PH 2.3.1 PH 2.3.2 PH 2.3.3
12. Historical and current global values An understanding of the past is necessary to solve the problems of the present. To grasp the necessity and urgency of planetary health, students need to be aware of the historical perspectives and milestones that have laid the foundation for the field, including those perspectives that have been historically marginalized or ignored. To identify opportunities for positive interventions, students must recognize patterns over time and appreciate current global context.	PH 1.2.5 PH 1.2.7 PH 1.2.8 PH 2.3.1 PH 2.3.2 PH 3.1.2

Third, the refined catalog of Planetary Health learning objectives was subjected to a first round of external peer review by two university professors for Global Health and Health and Climate Change in Germany, respectively. Based on their comments and feedback, we further refined the learning objectives.

Fourth, given that the Planetary Health learning objectives are part of the non-mandatory but cross-cutting chapters of the National Catalog of Learning Objectives, we established links to the mandatory core curriculum to showcase the relevance of Planetary Health to other medical subject areas. To this end we (1) implemented cross-references between learning objectives in the Planetary Health catalog and the core curriculum and (2) added examples pertinent to Planetary Health to learning objectives in the core curriculum. Initially, we identified learning objectives suitable for cross-linking and adding Planetary Health examples by a double-blinded screening of the core curriculum. Next, we reached consensus in pairs and the entire task force on which learning objectives were to be linked and the wording and location of Planetary Health examples to be included in the core curriculum, respectively. Finally, we sought approval from the chapter authors of the core curriculum for integrating Planetary Health examples in their chapters.

In 2021, the Institute for Medical and Pharmaceutical Examinations (*Institut für Medizinische und Pharmazeutische Prüfungsfragen*, IMPP), responsible for developing and administering centralized state examinations, established an interdisciplinary working group on *Climate, Environment and Health Impact Assessment* (22) coordinated by two members of the task force (EMS and ME). The working group aims at informing and leading on the development of Planetary Health-related exam questions to be included in state examinations for physicians and other health professionals and is comprised of experts with a broad spectrum of expertise including different medical specialties, psychology, psychotherapy, sociology, and transition research. In a fifth and final step, members of the working group reviewed the Planetary Health learning objectives and suggested further refinements based on several rounds of discussions within the working group. Refinements were mostly focused on concretizing content of specific learning objectives and examples. The final catalog of national Planetary Health learning objectives was published together with the core curriculum in July 2021 and is now available online (23). An English translation of the learning objectives is presented in Appendix 2 in the Supplementary material.

## 3. Conclusions and next steps

To our knowledge, the catalog of national Planetary Health learning objectives is the first attempt to operationalize general principles of PHE and to develop a catalog of actionable learning objectives for medical education in Germany. Following the principle of constructive alignment, defined here as the alignment of teaching, and learning activities and assessment tasks with intended learning objectives (24), we anticipate that the Planetary Health learning objectives will support the development and implementation of innovative Planetary Health teaching, learning and exam formats in medical schools. Complementing local initiatives in medical schools, the adaptation of national state examinations based on the catalog of Planetary Health learning objectives is a promising lever to stimulate PHE in medical schools, independent of local initiatives. The activities described in this article complement a small number of similar initiatives internationally (25–28). The availability of other PHE catalogs will provide opportunities for cross-country and interdisciplinary exchange of experiences and validation of content, thus supporting the consolidation of Planetary Health learning objectives and alignment of PHE for all health professionals.

Given the increasing health impacts of the climate and ecological crises (2), we expect a growing interest from policy and health systems stakeholders in actions to adapt to and mitigate these crises in the healthcare sector, in Germany and internationally. This is underscored, *inter alia*, by the adoption of several motions at the annual meeting of the German Medical Association, including a motion on the integration of Planetary Health in continuous professional education (29). PHE guided by the national Planetary Health learning objectives has the potential to equip physicians with the knowledge, values, skills, confidence, and leadership attributes to adequately respond to the climate and ecological crises. We are glad to share our experiences with interested individuals and organizations worldwide and are grateful for any feedback that stimulates the refinement of the current version of the national Planetary Health learning objectives for Germany.

## Data availability statement

The original contributions presented in the study are included in the article/Supplementary material. Further inquiries can be directed to the corresponding author.

## Author contributions

KW wrote the first draft of the manuscript. E-MS-S and ME provided substantial conceptual input to the manuscript. All authors critically reviewed the different versions of the manuscript, suggested revisions, and approved the version to be published.
