# Supplementary material for: National Planetary Health learning objectives for Germany: A steppingstone for medical education to promote transformative change
Source: Front Public Health. 2023 Feb 16;10:1093720. doi: 10.3389/fpubh.2022.1093720 (PMC10015604; doi:10.3389/fpubh.2022.1093720)
Supplement: Supplementary file 1 [file Table_1.docx]

Supplementary Material

**Appendix 1: Medical education and reform processes in Germany**

The Institute for Medical and Pharmaceutical Examinations (*Institut für Medizinische und Pharmazeutische Prüfungsfragen*, IMPP), founded in 1972, develops and delivers the centralized state examinations for students of medicine, dentistry, pharmacology, and psychotherapy based on the Medical and Pharmaceutical Licensing Regulations and the Law of Psychotherapy. Furthermore, it develops syllabi for the state examinations (*Gegenstandskataloge*, GK) and conducts medical education research focusing on examinations (Institut für Medizinische und Pharmazeutische Prüfungsfragen (IMPP) n/d)

The National Competency-based Catalogue of Learning Objectives for Medical Education (hereinafter referred to as National Catalogue of Learning Objectives; *Nationaler Kompetenzbasierter Lernzielkatalog Medizin*, NKLM) stipulates competencies covering different levels of learning including knowledge, attitudes, values and skills, medical students should have acquired by the end of their studies. The National Catalogue of Learning Objectives was developed under the auspices of the Association of Medical Faculties (*Medizinischer Fakultätentag*, MFT) that represents all 39 public medical schools in Germany.

The first version of the National Catalogue of Learning Objectives was published in 2015 without being mandatory for medical schools (Medizinischer Fakultätentag (MFT) n/d) and was revised in a three-year multi-stakeholder process steered by the Association of Medical Faculties and conducted in cooperation with the Institute for Medical and Pharmaceutical Examinations and the research group LOOOP (Learning Opportunities, Objectives and Outcomes Platform) at the Charité – Universitätsmedizin Berlin (Charité Universitätsmedizin Berlin n/a). The revised version is now available for public review (Medizinischer Fakultätentag (MFT) n/d) and will be further updated until adoption by the Federal Council. The revised version consists of a mandatory core curriculum and is accompanied by several non-mandatory but thematically cross-cutting chapters that can be covered by medical faculties. The mandatory core curriculum consists of 8 chapters. The first two chapters provide technical and background information. The remaining six chapters focus on theories and ideas of human beings, graduate profiles, reasons for consulting physicians, diseases, overarching learning objectives as well as overarching competencies. The non-mandatory cross-cutting chapters include medical sociology, diversity, and Planetary Health and feature a three-level structure comprised of competencies, sub-competencies and learning objectives.

The revised version of the National Catalogue of Learning Objectives will be included in the updated Medical Licensing Regulations (*Ärztliche Approbationsordnung*) planned to go into effect in 2025 (Medizinischer Fakultätentag (MFT) n/d). Together with the Medical Licensing Regulations, the National Catalogue of Learning Objectives will become the legal foundation for medical education in all medical schools in Germany and will be aligned with the syllabi for the state examinations developed by the Institute for Medical and Pharmaceutical Examinations. According to the current draft of the updated Medical Licensing Regulations (not publicly available), approximately 80% of teaching will be devoted to the mandatory core curriculum and 20% will target focus areas that medical schools will develop locally.

**References**

Charité Universitätsmedizin Berlin (n/d). "LOOOP - the non-commercial, international network for curriculum development and curriculum mapping." Retrieved 4th of November, 2022, from <https://looop.charite.de/en/>.

Institut für Medizinische und Pharmazeutische Prüfungsfragen (IMPP) (n/d). "Institut für Medizinische und Pharmazeutische Prüfungsfragen (IMPP)." Retrieved 31st of October, 2022, from <https://www.impp.de/start.html>.

Medizinischer Fakultätentag (MFT) (n/d). "Kompetenzbasierte Lernzielkataloge (NKLM, NKLZ) – aus den Fakultäten und für die Fakultäten." Retrieved 31st of October, 2022, from <https://medizinische-fakultaeten.de/themen/studium/nklm-nklz/>.

Medizinischer Fakultätentag (MFT) (n/d). "Masterplan Medizinstudium 2020: Fachwissen und Erfahrung der Universitäten in der politischen Planung stärker berücksichtigen." Retrieved 31st of October, 2022, from <https://medizinische-fakultaeten.de/themen/studium/masterplan-medizinstudium-2020/>.

**Appendix 2: National Planetary Health learning objectives**

| The catalog of national Planetary Health learning objectives is comprised of three overarching competencies (PH.1, PH.2, PH.3), reflecting the triad of knowledge, attitudes and values as well as leadership skills, eight sub-competencies and 31 learning objectives. Each learning objective is accompanied by several health-related examples. | | |
| --- | --- | --- |
| **ID** | **Competencies, sub-competencies and learning objectives** | **Health-related examples** |
| PH.1 | Graduates demonstrate foundational knowledge about core areas of Planetary and Global Health |  |
| PH.1.1 | They describe anthropogenic environmental changes and demonstrate understanding of associated health effects. They can ... |  |
| PH.1.1.1 | ... describe and critically reflect on important anthropogenic environmental changes. | climate change |
|  |  | air pollution |
|  |  | ocean acidification |
|  |  | depletion of freshwater resources |
|  |  | land-use change (e.g., through urbanisation, industrial agriculture) threatening soil health |
|  |  | loss of primary forests (e.g., Amazon, Bialowieza) |
|  |  | biodiversity loss/extinction of species |
|  |  | dysregulation of phosphorous/nitrogen cycles |
|  |  | destruction of the ozone layer |
|  |  | accumulation of microplastic in organisms and habitats |
|  |  | overfishing and exploitation of other natural resources |
| PH.1.1.2 | ... describe environmental changes as complex, non-linear phenomena and reflect on the effects of feedback loops and unforeseeable tipping points as well as the urgency of countermeasures. | concept of planetary boundaries (e.g., concentration of CO_2_ in the atmosphere as an indicator for climate change) |
|  |  | concept of tipping points (e.g., melting ice shields at poles with subsequent reduction of the albedo effect and non-linear acceleration of climate change) |
|  |  | urgency of measures to protect the climate and natural environment as well as to adapt to climate and environmental changes according to the precautionary principle |
|  |  | natural habitats as examples for complex-adaptive systems |
| PH.1.1.3 | ... analyse and critically reflect on the impact of human activities on environmental changes in the Anthropocene | greenhouse gas emissions and air pollution caused by motorised traffic and transport |
|  |  | greenhouse gas emissions and land-use change caused by diets containing a large share of animal products (e.g., methane emissions through industrial beef production, monocultures for fodder production) |
|  |  | greenhouse gas emissions caused by fossil-fuel based energy production |
|  |  | population growth |
|  |  | destruction of ecosystems through intensive agriculture and industrial animal production (e.g., loss of nutrients and soil erosion, loss of biodiversity) |
|  |  | antimicrobial resistances exacerbated by the excessive use of antibiotics in human and veterinary medicine |
|  |  | depletion of natural resources caused by overuse |
|  |  | anthropogenic radioactive emissions |
| PH.1.1.4 | ... describe the effects of environmental changes on human health and analyse their basic mechanisms. | living conditions in the Holocene versus the Anthropocene |
|  |  | increase of climate change-related morbidity and mortality related to heat waves and freshwater scarcity (e.g., cardiovascular diseases, respiratory diseases, renal failure, kidney stones, dehydration, reduced physical activity), extreme weather events (e.g., trauma, disruption of care for patients with chronic conditions, migration) and salination of groundwater through sea-level rise (e.g., hypertension) |
|  |  | changes in morbidity and mortality caused by infectious diseases related to spatial shifts of disease vectors (e.g., malaria, dengue fever, Lyme borreliosis, bartonella-associated infections, yellow fever, schistosomiasis, leishmaniasis, Chagas disease, Zika fever, Chikungunya fever, diarrhoeal diseases, respiratory infections, Hanta virus haemorrhagic fever) or inadequate water provision and sanitation in refugee camps following extreme weather events (e.g., diarrhoeal diseases, respiratory infections, chickenpox, cholera or measles outbreaks) |
|  |  | increased risk of zoonoses, specifically emerging infectious diseases, related to the fragmentation of habitats, climate change, biodiversity loss or changed exposure dynamics (e.g., Ebola virus, SARS-CoV, MERS, SARS-CoV-2) |
|  |  | increases morbidity and mortality due to respiratory diseases (e.g., asthma, COPD, lung cancer, lower respiratory tract infections) related to air pollution (e.g., fine particulate matter, sulphur oxides, nitrous oxides, ozone, smog) |
|  |  | increased morbidity and mortality due to cardiovascular diseases (e.g., coronary heart disease, cardiac arrythmias, heart failure, cerebrovascular diseases) related to air pollution (e.g., fine particulate matter, sulphur oxides, nitrous oxides, ozone, smog) |
|  |  | increase in metabolic diseases (e.g., diabetes mellitus) related to air pollution (fine particulate matter) |
|  |  | altered quality of sperms related to air and water pollution with adverse effects on reproductive health |
|  |  | increased incidence of seizures associated with infections, heat waves, and fine particulate matter |
|  |  | morbidity and mortality due to crop failures during droughts (e.g., anaemia, visual impairment, mental conditions), lack of proteins and micronutrients (e.g., iron, zinc, vitamin B (reduced content in crops at increased atmospheric CO_2_ levels), vitamin A, omega-3 fatty acids) and overnutrition caused by excess calorie intake and intake of ultra-processed foods (triple burden of malnutrition) |
|  |  | morbidity and mortality associated with migration and violent conflicts aggravated by environmental changes |
|  |  | increase in allergic diseases related to pollen |
|  |  | increase rates of dementia and child developmental disorders related to air pollution (fine particulate matter) |
|  |  | health risks for mothers and newborns during heat waves (e.g., increased risk for preterm birth, gestational hypertension, placenta insufficiency, pre-eclampsia, mechanical ventilation, meconium aspiration) |
|  |  | potential negative impact on personality development and increase of mental conditions (e.g., depressions, PTSD, substance abuse, peritraumatic stress disorder, anxiety, increased risk of suicide) related to environmental changes and the (anticipated) loss of habitats; distinction between mental illnesses and adequate psychological reactions in light of environmental change such as eco-anxiety, eco-grief and solastalgia; aggravation of pre-existing mental illnesses during heat waves (e.g., increased rates of suicide and admissions to psychiatric wards), increased mortality due to psychoses, dementia or substance abuse during heat waves |
|  |  | morbidity and mortality (e.g., stress, depression, anxiety, cardiovascular diseases, diabetes, respiratory diseases) related to urbanisation (e.g., air pollution, noise pollution, traffic accidents, sedentary lifestyle, inadequate housing, urban heat islands, restricted access to green space) |
|  |  | sleep disorders, particularly in urban areas, related to heat waves/tropical nights, noise and light pollution |
|  |  | increased morbidity (e.g., neuropsychiatric disorders, malignancies) related to metals (e.g., lead, cadmium, chrome, arsenic) |
|  |  | increased morbidity and mortality related to conflicts and migration caused or aggravated by environmental change (e.g., trauma, malnutrition and hunger, depression, anxiety, substance abuse, PTSD, increased child and maternal mortality, STDs, diarrhoeal diseases, respiratory diseases, typhus, hepatitis A and E, meningitis, cholera, measles outbreaks) |
|  |  | increase in UV-associated dermal lesions due to reduced ozone layer |
|  |  | morbidity and mortality due to triage following extreme weather events (e.g., floods, forest fires) |
|  |  | increase in hormone-related morbidity (e.g., non-Hodgkin lymphoma, obesity, insulin resistance, dyslipidaemia, hormone-sensitive malignancies such as breast, ovarian and prostate cancer, thyroid diseases, reproductive dysfunction, behavioural disorders) caused by endocrine disruptors (e.g., bisphenole A, pesticides, flame retardants) |
|  |  | morbidity in humans and animals cue to anthropogenic radioactive emissions |
| PH.1.1.5 | ... identify population groups particularly vulnerable to environmental changes and describe their vulnerability factors. | children (e.g., malnutrition, increased risk for allergies) and infants (e.g., heat, risk of dehydration) |
|  |  | new-borns and elderly (e.g., increased risk of dehydration) |
|  |  | pregnant persons (e.g., increased vulnerability to infectious diseases and heat, higher risk of pregnancy and birth complications) |
|  |  | women (vulnerability due to gender roles, experience of higher psychological strain, violence (gender-based violence, GBV), loss of access to sexual and reproductive health care after extreme weather events and climate-related migration) |
|  |  | individuals with chronic conditions (e.g., asthma, chronic kidney disease) |
|  |  | individuals on long-term medication (risk of adverse effects in case doses are not adapted during heat waves) |
|  |  | individuals vulnerable to mental conditions |
|  |  | individuals in precarious housing, working and living environments (e.g., dense buildings, restricted access to green spaces for social and sports activities, workplaces without shade/heat protection, noise exposure) |
|  |  | individuals from lower socio-economic backgrounds |
|  |  | migrants |
|  |  | populations living in regions primarily affected by the consequences of global environmental changes (e.g., Sub-Saharan Africa, coastal populations) |
|  |  | Populations living in regions without social welfare systems (e.g., health, accident, or unemployment insurance) |
|  |  | additive effects of different vulnerability factors (e.g., pronounced adverse effects on migrant women from lower socioeconomic backgrounds) |
| PH.1.2 | They describe core concepts and stakeholders in Planetary and Global Health. They can ... |  |
| PH.1.2.1 | ... describe the distribution of morbidity and mortality in different world regions, age and gender strata and in groups with different socioeconomic backgrounds. | global burden of disease including different associated metrics (e.g., DALY, QALY) |
|  |  | child and maternal mortality |
|  |  | infectious diseases |
|  |  | mental illnesses |
|  |  | non-communicable diseases and their four most important risk factors (alcohol, tobacco, malnutrition of all forms, lack of physical activity) |
|  |  | accidents, violence |
|  |  | demographic and epidemiologic transition |
| PH.1.2.2 | ... reflect on the distribution of morbidity and mortality by taking into account social determinants of health, health inequalities and health inequities. | health as a human right (duty of the state to protect, respect and ensure the right to health) |
|  |  | effects of discrimination on morbidity and mortality (e.g., gender, ethnic background) |
|  |  | access barriers to healthcare at local, national, and global level |
|  |  | pronounced effects of anthropogenic environmental changes on individuals with lower socioeconomic backgrounds |
|  |  |  |
| PH.1.2.3 | ... critically appraise research approaches and evidence in Planetary and Global Health | choice of research questions (e.g., drugs for neglected tropical diseases, NTDs) |
|  |  | choice of study populations (e.g., limited research on women’s health) |
|  |  | biases (e.g., selection bias, measurement bias) |
|  |  | unequal availability of evidence from different world regions (e.g., majority of studies conducted in the US and Europe) |
|  |  | critical appraisal of the quality of evidence using established quality appraisal tools |
|  |  | limited quantity and quality of evidence in Planetary and Global Health |
| PH.1.2.4 | ... describe and critically appraise essential characteristics, advantages and disadvantages of different healthcare systems in the context of Planetary and Global Health. | dimensions of access to healthcare (availability, accessibility, affordability, acceptability) |
|  |  | WHO building blocks of a healthcare system |
|  |  | different financing systems and associated healthcare costs (e.g., tax-based, fee-based, out-of-pocket payments) |
|  |  | sustainability of healthcare systems (e.g., divestment in medical pension funds, low-emission medical institutions) |
| PH.1.2.5 | ... reflect on the impact of globalization on health, healthcare systems and healthcare. | precautionary principle vs polluter-pays principle |
|  |  | global spread of diseases associated with trade and travel (e.g., during pandemics, spread of vectors and agents caused by global trade with plants and food) |
|  |  | health workforce migration and the associated brain drain |
|  |  | inequalities caused by global trade and investments |
|  |  | vulnerability of migrants |
|  |  | role of transnational trade concerning the availability, consumption, and marketing of harmful products |
|  |  | marketing by pharmaceutical companies and other corporations in the healthcare sector (e.g., private clinics) |
|  |  | privatization of medical supply services |
|  |  | increase in antimicrobial resistance |
|  |  | beneficial impacts of globalization on health (e.g., increased availability of and access to drugs and healthcare services) |
| PH.1.2.6 | ... name important actors in the context of Planetary and Global Health at the local, national, and supranational level and appraise their tasks and responsibilities. | supranational actors: United Nations (UN) and their affiliated organizations, especially WHO and other organizations relevant for Planetary and Global Health (e.g., FAO, UNICEF, UNFPA, UNEP, UNFCCC); the World Bank |
|  |  | private non-profit actors (e.g., foundations such as Bill and Melinda Gates Foundation or Wellcome Trust) |
|  |  | private for-profit actors (e.g., pharmaceutical or medical technology companies) |
|  |  | civil society organizations (e.g., Doctors Without Borders, Red Cross) |
|  |  | international alliances (e.g., Planetary Health Alliance, inVIVO Planetary Health Network, Consortium of Universities for Global Health, International Physicians for the Prevention of Nuclear War (IPPNW), People's Health Movement) |
|  |  | national alliances (e.g., Global Health Alliance Deutschland (GHA-D), Global Health Hub Germany (GHHG), German Alliance for Global Health Research (GLOHRA), German Alliance for Climate Change and Health (KLUG), Health For Future, Climate Psychology Alliance UK (CPA), Psychologists/Psychotherapists For Future Germany) |
|  |  | science (e.g., Potsdam-Institute for Climate Impact Research, Wuppertal Institute) |
| PH.1.2.7 | ... describe important concepts and initiatives to reduce health inequalities and inequities as well as to improve Planetary and Global Health. | UN Convention on Human Rights |
|  |  | Millennium Development Goals (MDGs) and Sustainable Development Goals (SDGs) |
|  |  | development cooperation and humanitarian aid |
|  |  | Ottawa Charter for Health Promotion |
|  |  | Lancet Commission on Health and Climate Change, Lancet Countdown on Health and Climate Change, EAT-Lancet Commission |
|  |  | Universal Health Coverage |
|  |  | Health in all Policies |
|  |  | Club of Rome |
|  |  | Conference of Parties (COP) |
|  |  | social, commercial, political, and economic determinants of health |
|  |  | International Health Regulations (IHR) |
|  |  | climate justice |
|  |  | Doughnut Economics (safe operating space for human activities within planetary boundaries based on social and health equity) |
|  |  | social-ecological transformation |
|  |  | ecological, social, and economic sustainability |
|  |  | resilience (e.g., sense of coherence, mindfulness, emotional reflective methods) |
|  |  | biophilia |
|  |  | governance (regulatory and steering mechanisms, *inter alia*, to implement international agreements) |
|  |  | systems thinking, complexity science, Earth systems science |
|  |  | leverage points, social tipping points |
|  |  | ecological footprint, political handprint |
|  |  | efficiency, consistency, and sufficiency as sustainability strategies |
|  |  | Peace through Health/Medical Peace Work |
| PH.1.2.8 | ... explain and critically reflect on the historical origins of Planetary and Global Health and power relations in historical and current contexts | Primary Health Care / Alma Ata Declaration |
|  |  | roots of tropical medicine and development cooperation in colonialism |
|  |  | differences and overlaps between Public - International - Global - One - Eco - Planetary Health |
|  |  | links between neo-colonialism and Global Health |
| PH.2 | Graduates reflect on their responsibility to maintain and foster health and the natural and societal systems on which it depends and demonstrate relevant competencies. |  |
| PH.2.1 | They describe areas requiring transformative change to enable health within planetary boundaries and identify concepts and actors to implement transformative change processes. They can ... |  |
| PH.2.1.1 | ... identify needs for transformation in different societal sectors necessary for healthy lives within planetary boundaries. | food system transition |
|  |  | mobility transition |
|  |  | land-use and agricultural transition |
|  |  | energy transition |
|  |  | economic and consumption transition |
|  |  | transition towards sustainable healthcare |
|  |  | changes in legal frameworks and interpretation of existing legislation |
|  |  | transformation of societal norms and values |
|  |  | transformation of political and fiscal conditions |
| PH.2.1.2 | ... describe the potential of interventions with positive effects on both the environment and human health (interventions with co-benefits). | sufficiency-oriented interventions such as expanding cycling and pedestrian infrastructure, transformation of parking spaces into public green spaces, fare-free public transport systems |
|  |  | expansion of renewable energy production (e.g., solar and wind energy) reduces air pollution and associated diseases (e.g., respiratory, cardiovascular) |
|  |  | expansion of urban green spaces (e.g., parks) reduces the urban heat island effect and improves psychological wellbeing |
|  |  | promotion of active transport (e.g., expansion of cycling and pedestrian infrastructure) reduces air pollution and lifestyle-related morbidity (e.g., metabolic syndrome) |
|  |  | expansion of e-mobility reduces air pollution and associated diseases (e.g., respiratory, cardiovascular) |
|  |  | promotion of predominantly plant-based diets (e.g., flexitarian) reduces habitat fragmentation amongst others and diet-related morbidity (e.g., diseases associated with the metabolic syndrome) |
|  |  | maintenance of natural habitats (e.g., agroforestry, regenerative agriculture) - reduces biodiversity loss and the risk of zoonoses |
|  |  | circular economy reduces chemical pollution and associated morbidity (e.g., exposure to endocrine disruptors and microplastic) |
|  |  | energy efficient and health promoting housing reduces greenhouse gas emissions and housing-related morbidity (e.g., through moist and cold) |
| PH.2.1.3 | ... characterize institutions such as kindergartens, schools, companies, or associations as important settings for transformative change processes. | healthy and sustainable communal catering (e.g., school or company cafeterias) |
|  |  | integration of values and attitudes relevant to Planetary and Global Health (e.g., ambiguity tolerance) in education for all levels |
|  |  | resilience based on individual self-efficacy and development |
|  |  | resilience based on collective and participatory efficacy and co-creation of infrastructures |
| PH.2.1.4 | ... describe municipalities as favourable settings for transformative change. | transformation of infrastructure for active mobility (e.g., expansion of cycling lanes and walkways whilst reducing space dedicated to motorised transport) and increased public transport to empower vulnerable groups |
|  |  | expansion of municipal green spaces |
|  |  | implementation of comprehensive urban development concepts to foster Planetary Health (e.g., doughnut economics in Amsterdam, Netherlands since 2020) |
|  |  | Deprivilege non-sustainable behaviors and structures |
| PH.2.2 | They reflect on core ethical principles of Planetary and Global Health and their role as future physicians. They can ... |  |
| PH.2.2.1 | ... describe ethical principles necessary for supporting and implementing transformative change based on physicians' responsibility for environmental and health protection and considering social determinants of health and cultural aspects. | Declaration of Geneva, concepts of medical ethics (e.g., patients' autonomy) |
|  |  | adequate ethical principles and moral attitudes for the Anthropocene (e.g., according to the Planetary Health Pledge (primum non nocere regarding people and planet, inter- and intragenerational justice, precautionary principle)) |
|  |  | reflection on and declaration of conflicts of interest |
|  |  | structural discrimination |
|  |  | climate justice (discrepancy between population groups that contribute and have contributed disproportionately to environmental changes and those that are and will be disproportionately affected by these changes) |
|  |  | distributive justice |
|  |  | ethical and legal conditions for research with vulnerable population groups in Germany and globally |
|  |  | special role of healthcare professionals based on society's trust in them and the responsibility for contributing to transformative change that arises thereof |
| PH.2.2.2 | ... describe roles of physicians necessary for supporting and implementing transformative change based on physicians' responsibility for environmental and health protection and considering social determinants of health and cultural aspects. | role of physicians as communicators |
|  |  | role of physicians as health advocates |
|  |  | role of physicians as leaders |
|  |  | role of physicians as medical experts |
|  |  | role of physicians as scholars |
|  |  | role of physicians as collaborators and team members |
|  |  | role of physicians as professionals |
|  |  | role of physicians as visionaries |
|  |  | physicians as change agents for transformative change processes |
|  |  | physicians as teachers including peer-teaching |
|  |  |  |
| PH.2.3 | They demonstrate intercultural competencies based on reflections on their own cultural, social, economic and educational background and professional position. They can ... |  |
| PH.2.3.1 | ... critically reflect on their motivation in the context of study-related stays abroad (e.g., internships, electives), their aims, the attainability of these aims as well as ethical dilemmas. | principle of *primum non nocere* (first do no harm) |
|  |  | identification and scrutiny of own privileges (e.g., critical whiteness) |
|  |  | avoidance of "voluntourism" |
|  |  | reflection on the ecological footprint of study-related stays abroad |
| PH.2.3.2 | ... apply the principle of *primum non nocere* (first do no harm) during study-related stays abroad in countries of the global South and carry out their activities in an ethically, culturally, and socially sensitive and reflexive manner. | consideration of medical standards independent of location or socio-cultural contexts |
|  |  | reflection on geographical, economic, and socio-cultural contexts before, during and after study-related stays abroad |
| PH.2.3.3 | ... are responsive to different concepts of health and disease as well as social, legal, linguistic, structural and cultural aspects of care when working with migrants whilst avoiding stereotyping. | medical pluralism |
|  |  | structural and legal determinants of health (e.g., residence status) |
|  |  | health of undocumented migrants |
|  |  | racism in the context of medical care including unconscious and conscious biases |
|  |  | stigmatization |
|  |  | experiences of discrimination |
| PH.2.3.4 | ... factor travel and migration histories, heterogeneous disease prevalences and incidences in different countries and heterogeneous determinants of health into differential diagnoses. | under-diagnosis of malaria caused by neglecting travel histories |
|  |  | over-diagnosis of HIV in people from Sub-Saharan Africa due to stereotyping |
|  |  | under- and mis-diagnosis of tuberculosis or leprosy |
| PH.3 | Graduates describe and demonstrate skills to stimulate and implement transformative change in healthcare and other sectors of society. |  |
| PH.3.1 | They demonstrate the capability to implement transformative change processes and to establish necessary preconditions. They can ... |  |
| PH.3.1.1 | ... describe the relevance of interprofessional and transdisciplinary cooperation for solving complex whole-systems problems in the context of Planetary and Global Health and demonstrate skills to initiate cooperation. | implementation of sustainable and health-promoting housing and urban development promoted by cooperation between health professionals, city planners, engineers, architects, and local politicians |
|  |  | implementation of sustainable food production and health-promoting diets promoted by cooperation between health professionals, agricultural scientists, farmers, and local politicians |
|  |  | process optimisation in healthcare institutions promoted by cooperation of management and health professionals of all disciplines |
|  |  | implementation of Health in all Policies approaches on the local, national, and global level |
| PH.3.1.2 | ... critically appraise different knowledge systems in context of environmental changes and their adverse health effects and apply those in the implementation of transformative change processes. | principles of physical therapy in the context of health promotion and prevention (e.g., Balneo and climate therapy) |
|  |  | traditional indigenous knowledge systems and resilience factors in different cultural contexts |
|  |  | traditional Chinese medicine (TCM) as an example for a healing concept from a different cultural context which has been integrated into Western medicine |
| PH.3.1.3 | ... name important inter- and transdisciplinary approaches and apply them in the implementation of transformative change processes considering local empowerment. | transdisciplinary research (e.g., real-world laboratories) |
|  |  | participatory health research (e.g., collaborative development of a climate protection plan by staff of a medical care center) |
|  |  | citizen involvement in the implementation of transformative change processes |
| PH.3.2 | They demonstrate skills for implementing transformative change processes in inter- and transdisciplinary settings. They can ... |  |
| PH.3.2.1 | ... explain principles of effective communication to promote the implementation of transformative change processes and demonstrate practical skills to apply them. | four levels of communication (Schulz von Thun) |
|  |  | five axioms of communication (Watzlawick) |
|  |  | motivational interviewing |
|  |  | de-bunking (intervention to decrease susceptibility for disinformation) |
|  |  | integration of solutions with co-benefits in medical consultations (e.g., active transport with positive effects on health and the environment, healthy and sustainable diets) |
|  |  | agenda setting, advocacy in conversations with decision-makers |
|  |  | strategies for science communication |
|  |  | use of medical metaphors to describe the effects of environmental changes on health (e.g., multi-organ-failure on our planet when several planetary boundaries are simultaneously transgressed) |
| PH.3.2.2 | ... integrate aspects of sustainability in medical consultations. | communication skills to differentiate rational from psychopathological reactions to the climate crisis, resource-activating communication style to increase resilience |
|  |  | address behaviors with positive effects on health and the environment (co-benefits, e.g., active transport, sustainable diets) |
|  |  | address whole-systems approaches for collective health, climate and environmental protection to increase self-efficacy, resilience and participation (e.g., changing societal and political conditions in local communities) |
| PH.3.2.3 | ... explain principles of effective leadership for transformative change processes and demonstrate skills to implement them in practice. | change management |
|  |  | collaborative/distributive leadership |
|  |  | sustainable development of organisations (e.g., Eco-Management and Audit Scheme (EMAS)) |
| PH.3.3 | They describe approaches to implement transformative change processes in healthcare systems and patient care. They can ... |  |
| PH.3.3.1 | ... identify and implement adaptation and mitigation measures in healthcare institutions and patient care. | reduction and safe disposal of environmentally harmful waste |
|  |  | increase of energy efficiency in healthcare institutions |
|  |  | use of sustainable energy sources in healthcare institutions |
|  |  | reduction of water use in healthcare institutions |
|  |  | promotion of predominantly plant-based, sustainably sourced diets for patients and staff of healthcare institutions (e.g., cafeterias) |
|  |  | strengthening of low-resource primary health care |
|  |  | reduction of healthcare-related mobility (e.g., expansion of telemedicine services) |
|  |  | implementation of climate-sensitive anticipatory guidance in well-child visits and health check-ups |
|  |  | addressing the health effects of environmental changes in history-taking, diagnostics, therapy, and prevention |
|  |  | consideration of vulnerable population groups in the context of environmental changes |
|  |  | reduction and recycling of valuable waste including glass, plastic, paper, and metals |
|  |  | offering e-mobility options to staff of healthcare institutions (e.g., e-bikes, e-cars), promoting public transport (e.g., provision of job tickets), improving infrastructure for cyclists in healthcare institutions (e.g., bike parking) |
| PH.3.3.2 | ... identify and implement adaptation and mitigation measures in their daily lives, research, future work environments and political decision-making. | public health (e.g., development and implementation of municipal heat action plans, expansion of municipal green spaces) |
|  |  | research (e.g., development and evaluation of measures that promote sustainability and health, transformative health research, transformation science) |
|  |  | political consulting (e.g., focus on environmental changes and their health effects as well as on policies that foster both health and sustainability) |
|  |  | science communication and knowledge translation (e.g., implementation of heat action plans in municipalities) |
|  |  | divestment in medical pension funds |
